# Validating National Kriging Exposure Estimation

**DOI:** 10.1289/ehp.10205

**Published:** 2007-07

**Authors:** Adam A. Szpiro, Lianne Sheppard, Paul D. Sampson, Sun-Young Kim

**Affiliations:** University of Washington, Seattle, Washington, E-mail: aszpiro@u.washington.edu

In their article Liao et al. (2006) argued that it is feasible to use national scale daily kriging to estimate ambient air pollution exposure, even in locations where monitoring data are limited. In addition, they argued that national scale kriging is preferable to regional kriging and that automated variogram estimation is preferable to manual. The advocated methodology seems appealing when compared with the more standard approach of estimating ambient exposure separately in individual metropolitan cities (Dockery et al. 1993; Jerrett et al. 2005; Miller et al. 2007; Pope et al. 1995) because it simplifies exposure assessment for multicity studies and allows inclusion of subjects far from monitoring sites. Liao et al. (2006) also suggested estimating daily variograms without accounting for day-today relationships and variations in data availability. This is a convenient simplification if it produces reliable results, but the evidence is not convincing.

The primary focus of the article by Liao et al. (2006) is on kriging daily ambient PM_10_ (particulate matter with aerodynamic diameter ≤ 10 μm) based on the U.S. Environmental Protection Agency Air Quality System (AQS) measurements. Three cross-validation statistics were reported, namely prediction error (PE), standardized prediction error (SPE), and root mean square standardized (RMSS). PE is the difference between predicted and measured concentrations at each site; SPE is the PE divided by the estimated SE; and RMSS is the SD of the SPEs across sites. PE and SPE can be regarded as measures of bias, and RMSS is a measure of the accuracy of the SE estimates (RMSS should be near 1, with RMSS > 1 indicating that the estimated SEs are too small). Cross-validation SE statistics were not reported, but the SE at Women’s Health Initiative (WHI) subject addresses is reported for some models.

The goal of kriging is accurate predictions at locations without measurements. This could be verified by a cross-validation mean square error (MSE) or similar summary of unsigned prediction error. In lieu of this, a reasonable alternative is to examine SE and RMSS together. If the RMSS is near 1 (< 1) then it is reasonable to regard the mean estimated SE as a valid estimate (upper bound) for the MSE. However, Liao et al. (2006) did not always report both the RMSS and SE, and some of their conclusions are erroneously supported by only one of these. We also note that limiting cross-validation to AQS sites may not be representative of performance at subject addresses.

The primary claim of Liao et al. (2006) is that the “data support the overall validity of kriging-based estimation approaches to estimate location-specific PM concentrations across the contiguous United States.” The authors argued that the average cross-validation PE and RMSS statistics are “acceptable” for 95% of days. However, PE and RMSS alone do not provide a reliable estimate of the prediction accuracy. An RMSS near 1 suggests that the SE is a good estimate of prediction accuracy, but the cross-validation SE was not reported. In another section of the article, the daily mean SE of predicted PM_10_ at WHI subject locations was reported to be 27.35 μg/m^3^, which is high compared with the overall mean concentration of 26.29 μg/m^3^.

Liao et al. (2006) claimed that national kriging is preferable to regional kriging, and they compareed their national model to one in which the continental United States is divided into five regions. They reasoned that the two models perform equally well under cross-validation based on comparisons of SPE and RMSS, so other issues such as missing data and locations near regional boundaries argue for a national approach (we note that the boundary issue is easily addressed by overlapping regions). However, because they did not report SE for the regional model, it is impossible to verify their claim that the national model performs equally well.

Finally, Liao et al. (2006) claimed that automated variogram estimation is preferable to manual. Based on 6 days of data, the authors argued that the manually fit model is worse because it produces somewhat larger SEs. However, the RMSS values on these days for the automatically fit model were all > 4, whereas they were near 1 for the manually fit model. Thus, comparing SEs to assess model accuracy is not valid because the SEs for the automatically fit model are unreliable. In fact, because the SEs were fairly similar and the RMSSs were significantly larger for the automatic fit, we would be inclined to favor the manual fit.

In summary, the methodology proposed by Liao et al. (2006) for national kriging would be appealing if it could be shown to be reliable. However, the reported statistics are not convincing.
